# Dealing with adverse drug reactions in the context of polypharmacy using regression models

**DOI:** 10.1038/s41598-024-78474-4

**Published:** 2024-11-09

**Authors:** Jakob Sommer, Roberto Viviani, Justyna Wozniak, Julia C. Stingl, Katja S. Just

**Affiliations:** 1 Institute of Clinical Pharmacology, University Hospital of RWTH Aachen, Wendlingweg 2, D-52074 Aachen, Germany; 2 Department of Radiology and Biomedical Imaging, Yale University School of Medicine, New Haven, CT USA; 3 Institute of Psychology, University of Innsbruck, Innsbruck, Austria; 4 Psychiatry and Psychotherapy Clinic III, University of Ulm, Ulm, Germany

**Keywords:** Adverse drugs reaction, Horseshoe, Lasso, Regression models, Polypharmacy, Geriatrics, Drug safety, Pharmacology, Computational models, Databases, Statistical methods, Virtual drug screening

## Abstract

Polypharmacy in older adults increases the risk of adverse drug reactions (ADRs), but studying this relationship is complex. In real-world data, the high number of medications, coupled with rare drug combinations, results in high-dimensional datasets that are difficult to analyze using conventional statistical methods. This study applies horseshoe and lasso regression for analyzing rare events in polypharmacy contexts, focusing on severe ADRs such as falls and bleedings. These regression models are executed on a multi-center dataset compiling 7175 cases from the ADRED project to detect potential ADR-associated drugs among 100 most common drugs in emergency department admissions. Positive predictors are classified by using 50% and 90% credibility intervals. This study demonstrates that regression models with horseshoe or lasso priors are effective for analyzing ADRs, providing a comprehensive consideration of multiple factors in large, sparse datasets and improving signal detection in polypharmacy, addressing a significant challenge in pharmacovigilance. Both priors yielded consistent and clinically meaningful results. The horseshoe regression resulted in fewer potential positive predictors overall, which could make it suitable as a diagnostic tool. While these regressions generate valuable information, there are still challenges in setting appropriate thresholds for determining and interpreting the positive results.

**Supplementary Information:**

The online version contains supplementary material available at 10.1038/s41598-024-78474-4.

## Introduction

Polypharmacy is common in multi-morbid and older patients as multiple diseases necessitate treatment with different drugs at the same time^1^. There are multiple sources mentioning a correlation between adverse drug reactions (ADRs), age, and polypharmacy^2,3^. With the higher age of people, numbers of drugs increase along with the risk for potentially serious ADRs^4^. A high number of drugs can result in pharmacokinetic or pharmacodynamic drug-drug interactions (DDIs) adding to age-related morbidity resulting in ADRs^5^. Patients with ADRs experience common consequences of ADRs like increased morbidity and mortality, leading to extended hospital stays and increasing the costs up to 1.9 times^6^. Meanwhile, a substantial proportion of ADR-related hospitalizations could be prevented^7,8^.

Falls or bleedings may serve as easily detectable ADRs occurring in older adults with polypharmacy^9,10^. There is a high number of studies analyzing drug-associated ADRs, such as falls or bleedings, but with heterogeneous, mostly observational study designs and often focusing on a certain drug or drug class, not respecting the influence of the whole medication^11–14^. Thus, it can be difficult to conclude how a drug treatment should be modified in an older adult^15^.

While studies often show older, multi-medicated adults affected by ADRs, the context of polypharmacy is only poorly addressed in these studies, and it is often impossible to identify a specific drug as responsible for the ADR^1^. A common approach to test the association between drugs and ADRs is to evaluate many models, one per drug, with the ADR as the outcome variable. In polypharmacy, the use of one drug may be associated in the sample with the use of other drugs, and pharmacokinetic and –dynamic DDIs may also provoke ADRs.

The problem becomes apparent in the use of real-world data for signal detection^16,17^. Transforming drugs into a matrix for regression analysis usually leads to several hundred rows per subject with rare events for every single drug and drug combination. What essentially is called sparsity is particularly relevant in the pharmacological context, where a high number of predictors relative to the sample size is common. With only a small set of drugs responsible for ADRs, many coefficients can be considered irrelevant. Conventional generalized linear models, such as logistic regression are well-established and easily interpretable^18^, but they are generally not suited for analyzing many predictors with rare binary outcomes, as this often results in overfitting and poor predictive performance^19^, and eventually leads to under-reporting^20^ or highly variating numbers for the prevalences of ADRs^21^. Thus, there is a need for a more comprehensive and sophisticated analytical approach.

Compared to traditional logistic regression, Bayesian methods allow for a more comprehensive approach by using a prior to control sparsity and handling a larger number of coefficients and covariates. This offers a significant advantage for detecting drug-related ADRs, even in datasets with rare occurrences, making Bayesian models particularly useful for signal detection in safety surveillance^22^. The lasso regression, for example, has already been successfully applied in biomedical datasets^23,24^.

The horseshoe regression is a novel statistical technique which, to the best of our knowledge, has not been applied to pharmacological data. It is particularly promising due to its ability to handle sparse data^25^. The regression uses a horseshoe prior to shrink coefficients towards zero, thereby avoiding overfitting and reducing noise in data^26^. Additionally, the horseshoe prior can perform variable selection^27^ and has been shown to be robust to outliers^25^. The same is true for lasso regression, which can apply a double-exponential or Laplace prior to offer similar advantages^28^. Overall, this regression technique leverages the strengths of handling sparsity and performing variable selection while benefiting from the interpretability of a conventional generalized linear regression model, especially in comparison with machine learning methods^29^.

The objective of this analysis is to apply two of these regression models, horseshoe and lasso regression for a comprehensive event detection analysis of drug-ADR-relationships on a multi-center dataset.

## Methods

### Study database

The data utilized in this study was obtained from the ADRED-study (‘Adverse Drug Reactions in Emergency Departments’, ADRED; trial registration DRKS-ID: DRKS00008979). ADRED was a multi-center and prospective observational study. Data collected between 2015 and 2021 in six large emergency departments (ED) of tertiary care and academic teaching hospitals was used. All patients in this analysis agreed to participate and provided written informed consent. The ADRED-study was conducted in accordance with the Declaration of Helsinki and approved by the ethical committee of the University of Bonn (202/15). For more information on the study design and participant recruitment, please refer to the published works^2,30^. In brief, patient consultations to the ED that were considered drug-related after standardized causality assessment for ADRs^31^ by trained study personnel were subsequently enrolled in the study database. In previous analyses of this cohort, it became clear that the study cohort primarily consisted of older adults with multiple existing medical conditions and polypharmacy^2,30^. The database includes various details such as patient demographics, drugs administered including over-the-counter (OTC) drugs, and primary and secondary diagnoses.

### Compilation of data

All ADR symptoms observed in relation to drug exposure, as determined through the standardized WHO-UMC causality assessment system^31^ on ED admission were thoroughly documented in the database. Trained study personnel used the causality assessment to define symptoms as drug-related or non-drug-related. All symptoms seen in an at least possible relation to a drug were defined as ADRs according to pharmacovigilance guidelines^30,32^. Symptoms classified as ADRs were coded according to the MedDRA terminology on a low-level term level. These terms are summarized at a preferred term level, which is the standard level for pharmacovigilance analysis. Thus, we used the preferred terms for describing the outcome ADRs^33^. We analyzed falls diagnosed as ADR on ED admission and coded them as preferred term. In case of bleedings, we used the SMQs (Standardised MedDRA Query) to group all relevant preferred terms. To this end, we combined the SMQ “hematopoietic erythropenia” and “hemorrhage terms” to define bleeding ADRs.

### Data curation

The ADRED dataset consisted of 7967 cases, out of which 791 cases represent patients who made a subsequent visit to the ED. We excluded one case due to missing medication records. To ensure accuracy, we only analyzed the first presentation of each patient as subsequent visits may be affected by the treatment received during the initial visit. Consequently, we removed the second presentation, leaving us with a reduced dataset of 7175 cases.

The ADRED database contained 1627 different substances. These were documented by trained study personnel on ED admission. All drugs taken by the patient including OTC were documented. In the case of an unclear description of the drug, a term as specific as possible was documented (e.g. antibiotic or analgesic). Even with more sophisticated models, 1627 different variables could lead to overfitting and complicate data interpretation. Hence, we opted to include the 100 most frequently used substances. To this end, we calculated the mathematical optimum between the lowest amount of coefficient and the highest amount of the total sum of all used drugs. Consequently, we excluded substances that appeared less than 32 times throughout the dataset. As a result, we included 224 different drugs into the next step of the analysis.

Within these 224 different drugs, we excluded drug names that were too unspecific, such as those only documented as “NSAID” or “immunoglobulin”. Additionally, combination drugs were separated into their components, with a few exceptions. Specifically, we did not split combinations where one component is pharmacologically insignificant and primarily serves to enhance the effect of the main substance (e.g., “amoxicillin/clavulanic acid”). In such cases, we retained only the main substance (e.g., “amoxicillin/clavulanic acid” as “amoxicillin”, “oxycodone/naloxone” as “oxycodone”, “tilidine/naloxone” as “tilidine”) to increase the number of data points for the primary substance in the analysis. We removed combination drugs that hold low pharmacological relevance and/ or food supplements, such as “macrogol/potassium chloride/sodium carbonate/sodium chloride” or “calcium carbonate” or “potassium chloride” (Supplement 1). By sorting the resulting list of different substances by their frequency within the ADRED dataset, we ended up with a list of the top 100 substances for our final analysis. To account for the excluded drugs that were not included in the final dataset, we calculated the number of excluded medications for each case. Drugs not included in the analysis are shown in Supplement 2.

To adjust for potential pharmacokinetic DDIs, we assessed drugs for being a substrate or inhibitor of relevant cytochrome P450 (CYP) enzymes using the Drug Interactions Flockhart Table™^34,35^. To this end, we checked all drugs for being substrates or inhibitors of CYP3A4, 2D6, 2C19, or CYP2C9 as these enzymes are involved in the metabolism of the most important drugs^36^.

**Fig. 1:**
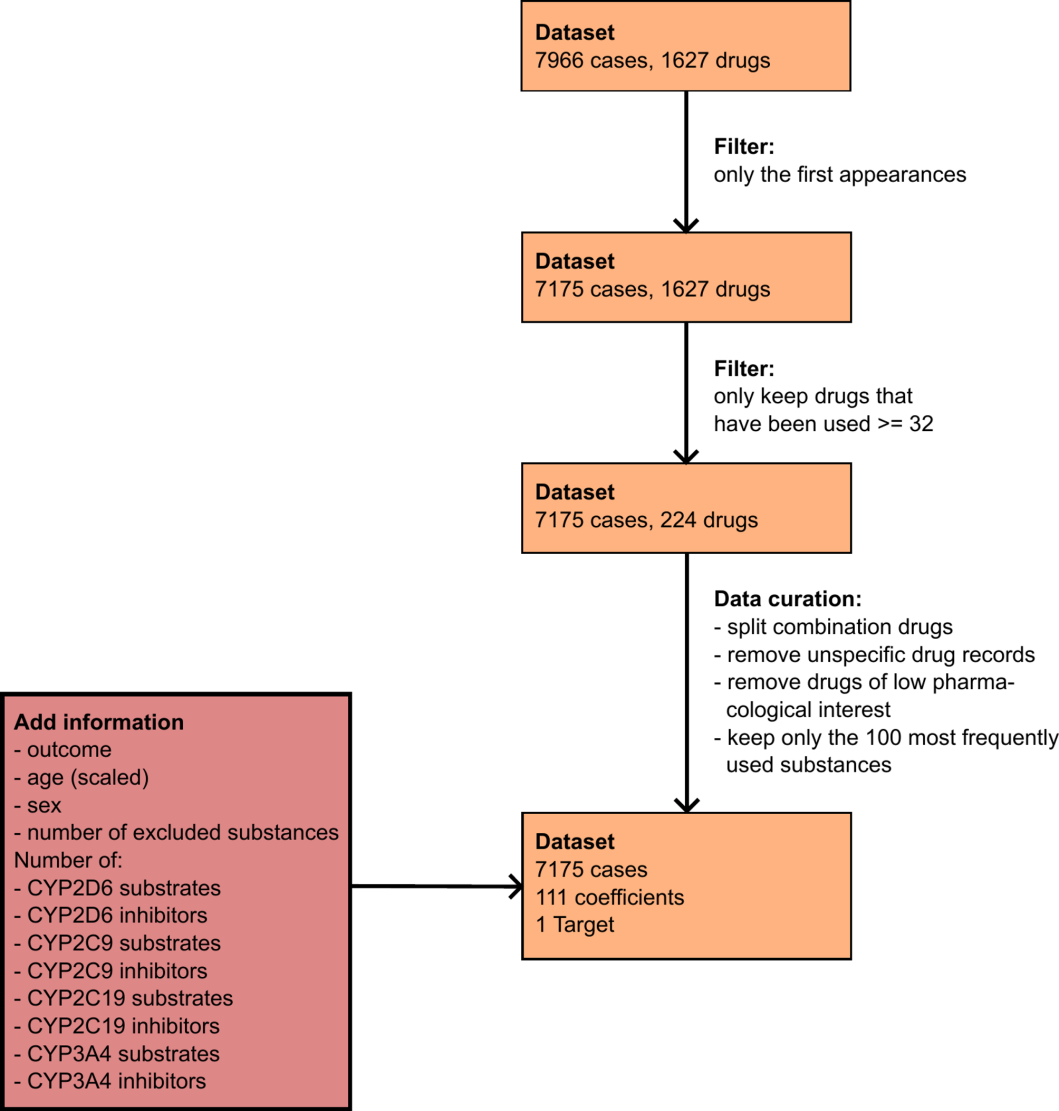
Flowchart of the dataset and data preparation for analysis.

Thus, we used patients’ basic information, including age (continuously in years), sex (male/ female), the calculated number of excluded medications per patient case, the number of CYP2C9, CYP2C19, CYP2D6, and CYP3A4 substrates, and inhibitors together with the 100 most common drugs in the final dataset. This resulted in a dataset size of 7175 × 112 (Fig. 1). We calculated models for the two chosen outcome ADRs “falls” and “bleedings” separately.

### Statistical analysis

Descriptive analysis was conducted for the final dataset to acquire medians and interquartile ranges (IQR) for continuous and absolute numbers and percentages for categorical variables. Logistic regression models with horseshoe and lasso priors were used to analyze the association of drugs with the occurrence of the outcome ADRs. The 100 drugs included in the analysis served as possible predictors. We defined the number of excluded drugs per case, age, sex, and the CYP substrates and inhibitors as relevant covariates to control for confounding effects.

To configure the regularized horseshoe regression model, we first set up a logistic regression (“family = binomial”) as a base model using the “stan_glm” function of the “rstanarm” library in the programming language R^37,38^. The stan_glm function allowed us to apply the hierarchical shrinkage prior right in the same step (“prior = hs()”). However, the parameter required some modifications: Following the explanation of Piironen and Vehtari^27^, we calculated the “global_scale” to be the “ratio of the expected number of non-zero coefficients to the expected number of zero coefficients, divided by the square root of the number of observations”^37^, essentially allowing a portion of the coefficients to escape the shrinkage. We expected a low number of influential predictors as most of the drugs in our list of predictors were by experience and literature review not inducing the outcome ADR. Therefore, we initialized the model through the global scale parameter for aggressive shrinkage, essentially allowing the model to be stricter in the identification of non-zero-coefficients. Now, using “slab_scale”, we were able to control how large the non-zero-coefficients are allowed to grow. We set the “slab_scale” as the square-root of the ratio of a constant and the number of non-zero-coefficients, then multiplied with the standard deviation of the outcome variable, thereby incorporating a sparsity assumption into the “slab_scale” value. The code is available under https://github.com/Fledermaus12/Horseshoe-Lasso-ADRED. Using these configurations, the hierarchical shrinkage prior strikes a balance between regularization and permitting data-driven influence on coefficient estimates. For the Bayesian lasso regression, we substituted the hs-formula with the “lasso”-formula in the “stan_glm” function to apply the Laplace prior.

Beta parameters, meaning the posterior distribution of regression coefficients, were analyzed for both models using the horseshoe and the lasso prior. 50% and 90% credibility intervals for each coefficient were analyzed and plotted in figures. The credibility intervals suggest that there is a minimum chance of 50%, respective 90%, that the true value of these predictors is non-zero. Therefore, if the posterior distribution of one drug does not cover zero with its 50%-interval, we classified the predictor as at least light positive. If the 90%-interval additionally does not cover the zero line, we classified the predictor as strong positive.

All statistical analyses were conducted with Python 3.7.11, Visual Studio Code 1.72, R 4.3.1, and R-Studio 2023.06.2.

## Results

The study cohort comprised older adults with a median age of 72 years (IQR 58; 81), in 50.8% (*n* = 3643) male sex, and with a median intake of 7 (3; 10) drugs.

In median 1 (0; 2) drug per patient case was excluded from the analysis due to rare intake as described above. In the fall analysis, *n* = 455 ADR cases of falls occurred and were compared to *n* = 6720 cases of other ADRs (no falls). In the bleeding analysis, *n* = 1977 ADR cases of bleeding occurred and were compared to *n* = 5198 cases of other ADRs (no bleeding).

Both horseshoe and lasso regression were successfully applied to the dataset to analyze associations between drugs and outcome ADRs in the context of polypharmacy. Some differences between the distribution of regression coefficients for drugs could be observed between the methods.

**Fig. 2:**
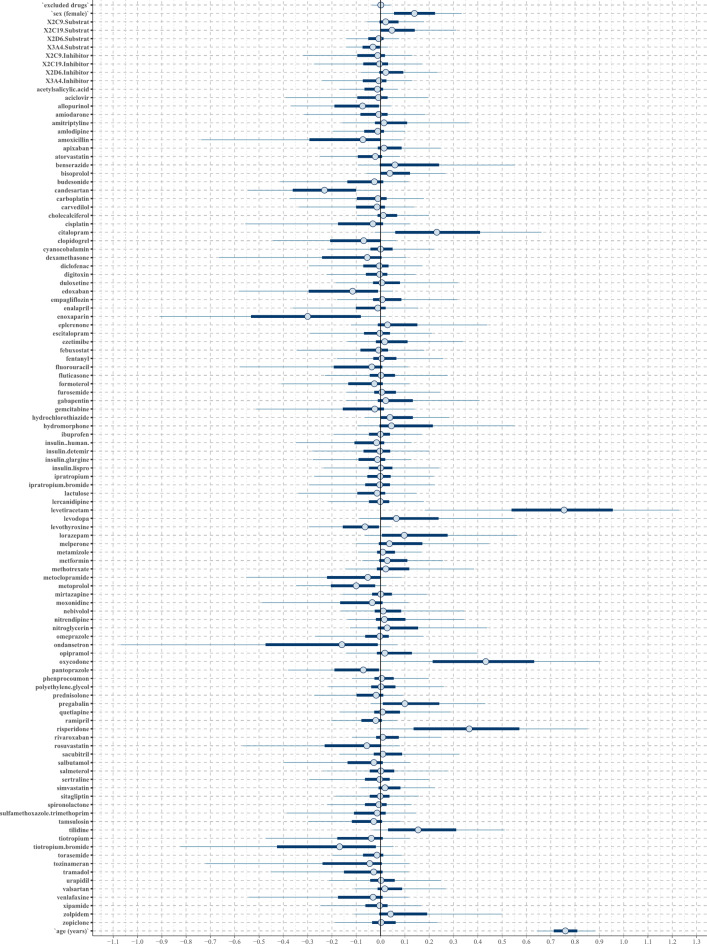
The posterior distribution of regression coefficients (beta parameters) in the horseshoe regression to the binary outcome fall. Each substance or confounder is represented in an individual row by a 50% interval (thick inner lines) and 90% interval (thinner outer lines), thereby showing the posterior distribution of each coefficient. If the 50% interval includes the black-marked zero-line at the center, this coefficient does not have any probable influence on the outcome fall.

**Fig. 3:**
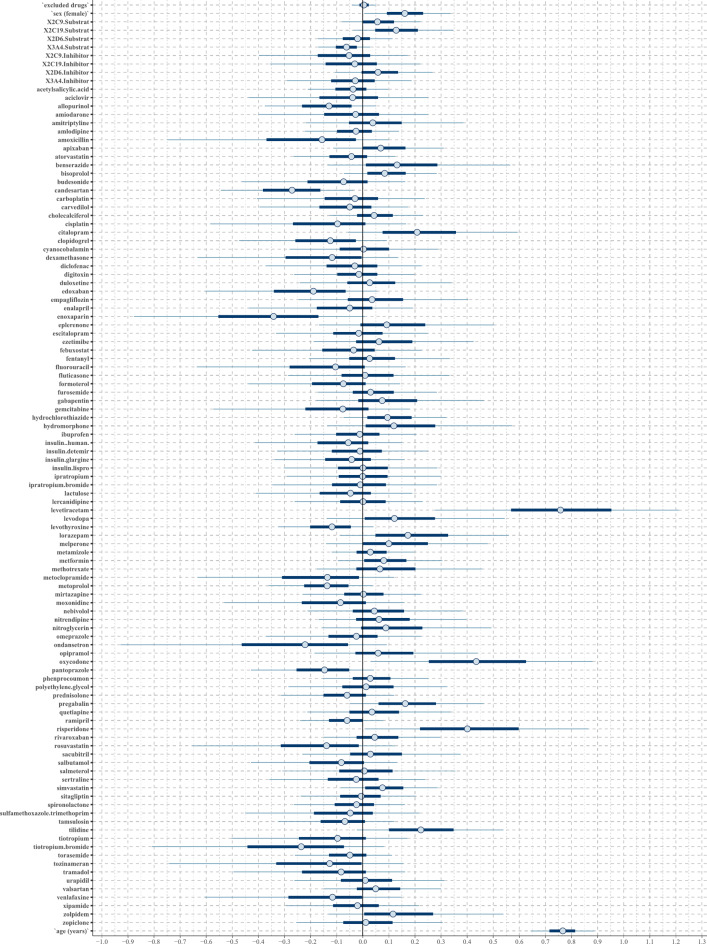
The posterior distribution of regression coefficients (beta parameters) in the lasso regression to the binary outcome fall. Each substance or confounder is represented in an individual row by a 50% interval (thick inner lines) and 90% interval (thinner outer lines), thereby showing the posterior distribution of each coefficient. If the 50% interval includes the black-marked zero-line at the center, this coefficient does not have any probable influence on the outcome fall.

In both regression models analyzing fall ADRs, the confounding factors age, female sex, and the number of CYP2C19 substrates showed positive associations (Figs. 2 and 3). Additionally, the lasso regression highlighted a strong positive tendency for female sex and a positive tendency for the number of CYP2C9 substrates taken.

Both models identified levetiracetam and oxycodone as strong positive predictors, while citalopram, tilidine, pregabalin, and lorazepam were recognized as light positive predictors (Table 1). Additionally, lasso regression highlighted risperidone as a strong positive predictor, whereas the horseshoe regression classified it as a light positive predictor. Lasso regression additionally identified hydrochlorothiazide, bisoprolol, zolpidem, levodopa, benserazide, simvastatin, metformin, and hydromorphone as light positive predictors. Negative predictors of both techniques are shown in Supplement 3.

**Table 1:** Potential positive predictors of regression methods for the fall ADR using horseshoe and lasso priors.

	Falls − 50% / 90% credibility intervals	
	Horseshoe	Lasso
Strong positive predictor		
Levetiracetam	**0.54–0.96 / 0.18–1.23**	**0.57–0.95 / 0.28–1.21**
Oxycodone	**0.21–0.63 / 0.00–0.90**	**0.25–0.63 / 0.03–0.88**
Risperidone	**-**	**0.22–0.60 / 0.01–0.86**
Light positive predictor		
Risperidone	**0.14–0.57** / -0.01–0.85	-
Citalopram	**0.06–0.41** / -0.02–0.66	**0.08–0.36** / -0.06–0.59
Tilidine/naloxone	**0.03–0.31** / -0.03–0.51	**0.03–0.31** / -0.03–0.51
Pregabalin	**0.01–0.24** / -0.04–0.43	**0.01–0.24** / -0.04–0.43
Lorazepam	**0.01–0.28** / -0.06–0.56	**0.05–0.33** / -0.09–0.56
Hydrochlorothiazide	-	**0.02–0.19** / -0.07–0.32
Bisoprolol	-	**0.02–0.16** / -0.07–0.28
Zolpidem	-	**0.01–0.27** / -0.13–0.54
Levodopa	-	**0.01–0.28** / -0.14–0.54
Benserazide	-	**0.01–0.29** / -0.14–0.57
Simvastatin	-	**0.01–0.16** / -0.08–0.29
Metformin	-	**0.01–0.17** / -0.10–0.31
Hydromorphone	-	**0.01–0.28** / -0.14–0.57

Credibility intervals that do not cover zero are shown in bold text.

**Fig. 4:**
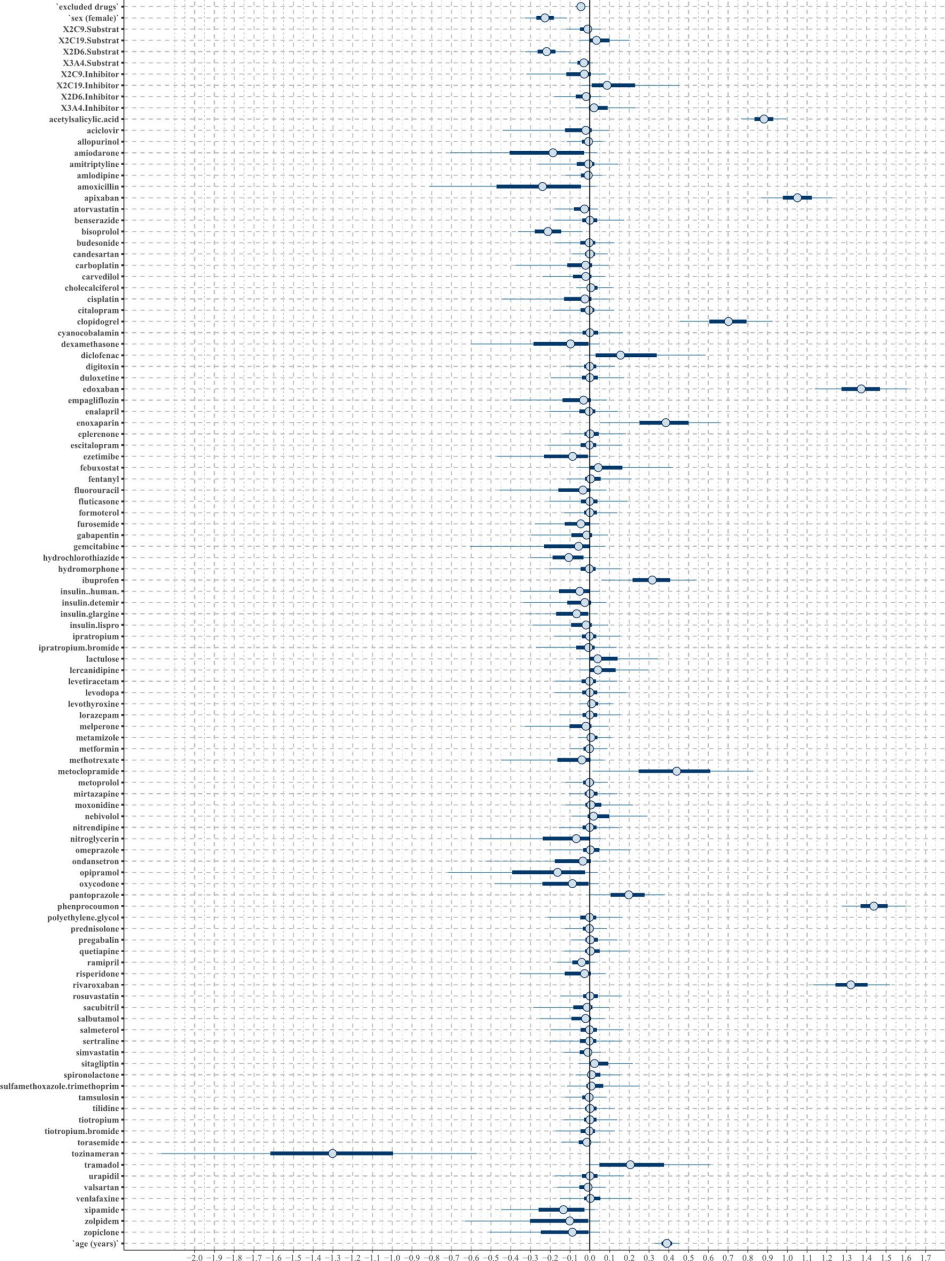
The posterior distribution of regression coefficients (beta parameters) in the horseshoe regression to the binary outcome bleeding. Each substance or confounder is represented in an individual row by a 50% interval (thick inner lines) and 90% interval (thinner outer lines), thereby showing the posterior distribution of each coefficient. If the 50% interval includes the black-marked zero-line at the center, this coefficient does not have any probable influence on the outcome bleeding.

**Fig. 5:**
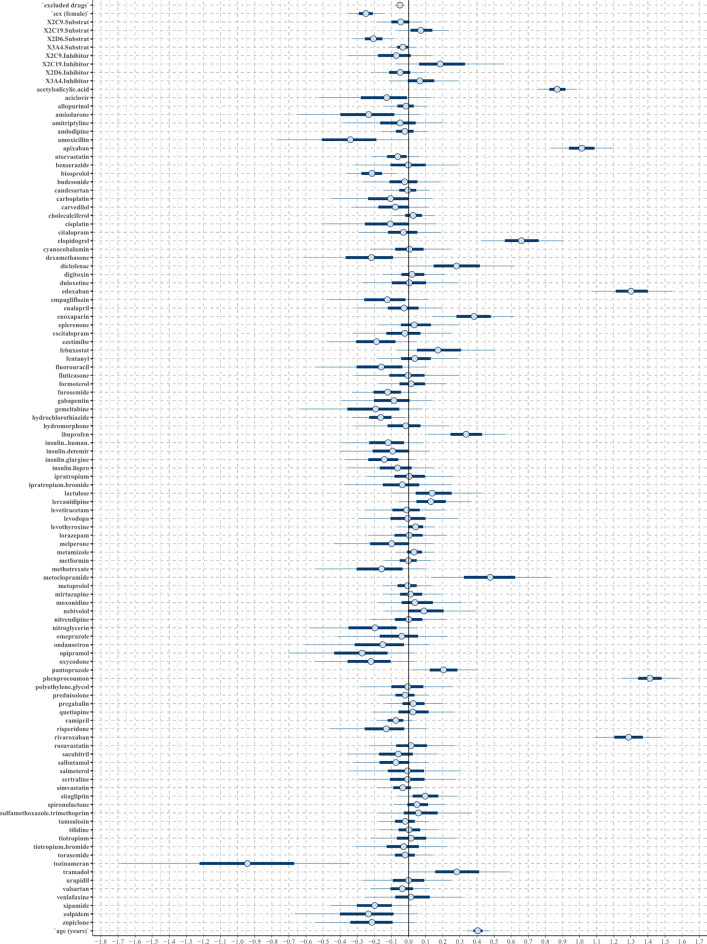
The posterior distribution of regression coefficients (beta parameters) in the lasso regression to the binary outcome bleeding. Each substance or confounder is represented in an individual row by a 50% interval (thick inner lines) and 90% interval (thinner outer lines), thereby showing the posterior distribution of each coefficient. If the 50% interval includes the black-marked zero-line at the center, this coefficient does not have any probable influence on the outcome bleeding.

Concerning bleeding ADRs, in both regression models, the confounding factor age showed a strong positive tendency, and the number of CYP2C19 substrates and inhibitors a light positive tendency (Figs. 4 and 5). As a difference, the lasso regression also exhibited a light positive tendency for the number of CY2C9 substrates.

Both regression models classified phenprocoumon, edoxaban, rivaroxaban, apixaban, acetylsalicylic acid, clopidogrel, metoclopramide, enoxaparin, ibuprofen, and pantoprazole as strong positive predictors and tramadol and diclofenac as light positive predictors (Table 2). Lercanidipine, lactulose, and sitagliptin are additionally classified as a light positive predictor by the lasso regression. Negative predictors are shown in Supplement 4.

**Table 2:** Potential positive predictors of regression methods for the bleeding ADR using horseshoe and lasso priors.

	Bleeding − 50% / 90% credibility intervals	
	Horseshoe	Lasso
Strong positive predictor		
Phenprocoumon	**1.37–1.51 / 1.28–1.60**	**1.34–1.48 / 1.24–1.58**
Edoxaban	**1.27–1.47 / 1.14–1.61**	**1.21–1.40 / 1.07–1.54**
Rivaroxaban	**1.24–1.41 / 1.13–1.52**	**1.20–1.37 / 1.09–1.48**
Apixaban	**0.98–1.12 / 0.87–1.23**	**0.94–1.09 / 0.83–1.20**
Acetylsalicylic acid	**0.83–0.93 / 0.77–1.00**	**0.82–0.92 / 0.75–0.99**
Clopidogrel	**0.60–0.79 / 0.46–0.93**	**0.56–0.76 / 0.42–0.91**
Metoclopramide	**0.25–0.61 / 0.01–0.83**	**0.32–0.62 / 0.13–0.83**
Enoxaparin	**0.25–0.50 / 0.05–0.66**	**0.28–0.48 / 0.14–0.62**
Ibuprofen	**0.22–0.41 / 0.06–0.54**	**0.25–0.43 / 0.11–0.57**
Pantoprazole	**0.11–0.28 / 0.00–0.38**	**0.12–0.29 / 0.02–0.40**
Light positive predictor		
Tramadol	**0.05–0.38** / -0.02–0.61	**0.16–0.41** / -0.00–0.59
Diclofenac	**0.03–0.34** / -0.03–0.59	**0.15–0.42** / -0.01–0.62
Lercanidipine	-	**0.05–0.22** / -0.06–0.36
Lactulose	-	**0.04–0.25 /** -0.10–0.43
Sitagliptin	-	**0.02–0.17 /** -0.06–0.28

Credibility intervals that do not cover zero are shown in bold text.

## Discussion

This study highlights the feasibility of using Bayesian regression models with horseshoe or Laplace priors for explorative analysis to detect potential drug candidates associated with a certain ADR. We were able to establish a much more comprehensive consideration of multiple factors in large and sparse datasets as it would have been possible with the more commonly used traditional logistic regression, which is known to overfit or even run into nonconvergence, especially in situations of high-dimensional data and rare events^39^. In situations close to nonconvergence one can observe coefficients taking very high non-sensible values^40^. It becomes necessary to apply the usage of variable selection or shrinkage parameters to eliminate overfitting, for example by applying priors to the regression formula^41^. Despite the usage of different priors, the horseshoe and lasso models produced consistent and clinically meaningful results, thereby confirming the effectiveness of both regression techniques.

The horseshoe regression’s shrinkage was able to reduce the long list of drugs to a small group of candidates, which previously was linked to ADRs in the literature. Considering the shrinkage properties, coefficients were naturally mostly centered around zero, though some tendencies remained evident. Similarly, lasso regression shrank most coefficients towards zero due to the Laplace distribution as well, but the shrinkage appeared to be less aggressive compared to the horseshoe model. This resulted in nearly twice the number of predictors for the fall ADR within the lasso model. Both methods showed greater consistency in identifying predictors for the bleeding ADR.

Overall, the horseshoe model applied stricter constraints than lasso regression. While lasso regression could serve as a broader screening tool, the horseshoe model appears to be more suited in refining the analysis to identify the strongest drug-ADR associations. This dual approach could enhance the accuracy and efficiency of detecting potential ADR-associated drugs and offer a practical and thorough method for prioritizing drugs for further investigation.

Among positive predictors in the fall analysis, we found several psychotropic drugs associated with falls, which is in line with conventional studies analyzing drug candidates^12^. However, in contrast to conventional methods, our models respected more drug variables as potential confounders. This results in a focus shift on drugs with currently less evidence than other drugs. Levetiracetam was the strongest predictor of fall among tested coefficients and positive in both horseshoe and lasso regression. It has been described in multiple sources to be fall-risk associated as well as causing symptoms related to falling such as somnolence, headache, asthenia, dizziness, and anorexia^42,43^. A meta-analysis even showed that somnolence and asthenia were dose-unrelated side effects with a low number needed to harm^44^, possibly indicating that even a small, prescribed dose within our dataset leads to more common falls. Citalopram also showed a strong positive tendency in our analyses and has been observed consistently with increased fall risk in other studies^12,45^. With respect to opioids such as oxycodone, there is also evidence suggesting an increased risk of falls^13^, which is consistent with known side effects such as dizziness and sedation. Furthermore, there is evidence that the use of opioids may increase the risk of falls, particularly in cases of polypharmacy^13^. Taken together, the data shows the plausibility of our results. The horseshoe regression resulted in a homogenous group of psychotropic drugs as positive predictors compared to the lasso, indicating a more conservative approach of the horseshoe technique.

The same is true for the bleeding analysis, where the models found multiple anticoagulant medications, including the direct oral anticoagulants (e.g. edoxaban, rivaroxaban, apixaban), vitamin K antagonist (e.g. phenprocoumon) and heparins (e.g. enoxaparin). Clopidogrel and acetylsalicylic acid, being antiplatelet agents, were also strongly associated with bleeding, as expected. Other results were ibuprofen (strong positive) and diclofenac (light positive), both being NSAIDs (non-steroidal anti-inflammatory drugs) and known to cause gastrointestinal bleeding and general risk of bleeding through indirectly affecting platelet aggregation^46^. Thus, with this outcome ADR, the models resulted again in highly plausible results. However, confounding by indication can also occur as expressed by metoclopramide and pantoprazole showing light associations with the bleeding ADR, as these drugs might be used in cases of gastrointestinal bleeding^47^.

By adding drug groups as potential predictors into our models, we can respect potential DDIs in our models, because associations of predictors with the outcome ADR are adjusted for each other. In addition, we considered potential pharmacokinetic DDIs by including metabolic pathways into our models. In all the models tested, we found an association of outcome ADRs with the CYP2C19 pathway. While, in the case of bleeding ADRs, this might already be explained by the frequent prescription of proton pump inhibitors in gastrointestinal bleeding^47^, there is evidence for fall ADRs, that the CYP2C19 pathway may be of importance for drug-associated falling^48^. However, this needs further analysis in hypothesis-driven approach studies.

Our analysis is subject to several important limitations that warrant careful consideration. First, our study cohort only comprises individuals who have all experienced ADRs. As often in pharmacovigilance studies, we need to compare a certain ADR (e.g. falls or bleedings) with a control group which consists of other ADRs^16,17^. This condition can lead to a leftward shift in the posterior distributions as an artifact, potentially obscuring coefficients that might otherwise be significant in comparisons such as ADRs versus non-ADRs, thus complicating the interpretation of the metrics. Notably, the identification of negative predictors cannot be generalized and interpreted as protective drugs. Next to falls, bleeding events are often documented in the dataset^2,4^. A decent number of patients in the non-fall cohort are also anticoagulant drug takers, explaining identified negative predictors. Another example is tozinameran in the bleeding analysis, which likely does not protect against bleeding but is associated with other side effects, making it more prevalent in the control group. Concerning negative predictors, our models reveal their exploratory nature, as drugs that are more commonly given in stable patients can also be found here.

The analysis benefits from a multi-center dataset, minimizing regional differences and hospital-specific confounding effects. This reduces noise and increases the representativeness of the findings. Consequently, our models show good performance and validity. However, the dataset lacked reliable information on the indications for which the drugs were prescribed and drug dosages. This problem arising could be observed in the case of levetiracetam associated with falls in our dataset or metoclopramide and pantoprazole associated with bleedings. In these cases, it is impossible to discriminate if the drug was prescribed because of already experienced symptoms connected with the final documented ADR or if the drug was the cause for the ADR. This issue could be solved by a conventional hypothesis-driven approach to control confounding by indication in future research.

An additional limitation arises from the choice of credibility interval. While the horseshoe prior offers good interpretability in general, the aggressive shrinkage applied in our model increases the difficulty of establishing reasonable thresholds to determine positive results, such as the 50%- or 90%-interval. While we have used the default settings for the interval provided by the software, the effects are not significant at conventional rejection thresholds of frequentist statistical models. This difficulty in setting appropriate thresholds for this Bayesian model may impede the practical application of the models and findings. In our analysis, any drug falling outside the 50% credibility interval is considered a potential cause of the ADR falls. However, altering the size of the credibility interval may yield varying results. This issue highlights the need for careful consideration when interpreting our findings. Additionally, a limitation arises from the fact that Bayesian models are not as widely adopted as frequentist statistical models. Some commonly used metrics in the frequentist framework, such as p-values, do not have direct equivalents in Bayesian frameworks. This disparity may cause confusion for users who are unfamiliar with Bayesian interpretation and pose an obstacle in accurately understanding these results.

Although we were able to include a much higher number of potential predictors into our models than usually analyzed, we still excluded drugs from our analysis. While we adjusted our models for the number of excluded drugs, potentially relevant drugs such as valproic acid, lamotrigine, ticagrelor, or doxazosin were excluded from our analyses. This is a clear limitation of our study.

Overall, our results suggest that the use of modified Bayesian regression models incorporating either horseshoe or lasso priors may be an effective approach to identify drugs associated with a certain ADR in a population with polypharmacy in exploratory research.

## Electronic supplementary material

Below is the link to the electronic supplementary material.

Supplementary Material 1

Supplementary Material 2

Supplementary Material 3

Supplementary Material 4

## Data Availability

The datasets analyzed during the current study are available from the corresponding author on reasonable request.
