# Supplementary material for: Dealing with adverse drug reactions in the context of polypharmacy using regression models
Source: Sci Rep. 2024 Nov 9;14:27355. doi: 10.1038/s41598-024-78474-4 (PMC11550797; doi:10.1038/s41598-024-78474-4)
Supplement: Supplementary file 1 — Supplementary Material 1 [file 41598_2024_78474_MOESM1_ESM.docx]

**Supplement 1**: List of substance modified or removed from the analysis due to expected low pharmacological relevance, overlap with food supplements or efficient grouping of similar substances.

| **name** | **command** | **rename** |
| --- | --- | --- |
| Amoxicillin/Clavulanic acid | rename | amoxicillin |
| Beclometasone/Formoterol | split |  |
| Benserazide/Levodopa | split |  |
| Budesonide/Formoterol | split |  |
| Calcium carbonate/Cholecalciferol | drop |  |
| Calcium carbonate/Colecalciferol (Vitamin D) | drop |  |
| Candesartan/Hydrochlorothiazide | split |  |
| Cinnarizine/Dimenhydrinate | split |  |
| Fenoterol/Ipratropium | split |  |
| Fluticasone/Salmeterol | split |  |
| Formoterol/Beclomethasone | split |  |
| Formoterol/Budesonide | split |  |
| Glycopyrronium bromide/Indacaterol | split |  |
| Hydrochlorothiazide/Ramipril | split |  |
| Insulin (human)/Insulin Isophane (human) | split |  |
| Potassium/Levothyroxine | split |  |
| Levodopa/Benserazide | split |  |
| Macrogol/Potassium chloride/Sodium carbonate/Sodium chloride | drop |  |
| Macrogol/Sodium chloride/Sodium bicarbonate/Potassium chloride | drop |  |
| Metformin/Sitagliptin | split |  |
| Naloxone/Oxycodone | rename | oxycodone |
| Naloxone/Tilidine | rename | tilidine |
| Olodaterol/Tiotropium | split |  |
| Ramipril/Hydrochlorothiazide | split |  |
| Sacubitril/Valsartan | split |  |
| Salmeterol/Fluticasone | split |  |
| Tilidine/Naloxone | rename | tilidine |
| ferrous cation | drop |  |
| folic acid | drop |  |
| magnesium | drop |  |
| potassium | drop |  |
| potassium chloride | drop |  |
