# Supplementary material for: Dealing with adverse drug reactions in the context of polypharmacy using regression models
Source: Sci Rep. 2024 Nov 9;14:27355. doi: 10.1038/s41598-024-78474-4 (PMC11550797; doi:10.1038/s41598-024-78474-4)
Supplement: Supplementary file 3 — Supplementary Material 3 [file 41598_2024_78474_MOESM3_ESM.docx]

**Supplement 3**: Potential negative predictors of regression methods using horseshoe and lasso priors for the outcome ADR “falls”

|  | **50%/ 90% credibility intervals** | |
| --- | --- | --- |
|  | **Horseshoe** | **Lasso** |
| **Light negative predictors** |  |  |
| allopurinol | **-0.19 – -0.01** / -0.37 – 0.04 | **-0.23 – -0.04** / -0.38 – 0.05 |
| clopidogrel | **-0.21 – 0.00** / -0.44 – 0.06 | **-0.26 – -0.03** / -0.47 – 0.09 |
| edoxaban | **-0.3 – -0.01** / -0.58 – 0.05 | **-0.34 – -0.07** / -0.60 – 0.06 |
| enoxaparin | **-0.53 – -0.08** / -0.91 – 0.02 | **-0.55 – -0.17** / -0.88 – 0.01 |
| levothyroxine | **-0.16 – -0.01** / -0.29 – 0.04 | **-0.2 – -0.05** / -0.32 – 0.04 |
| metoprolol | **-0.2 – -0.02** / -0.35 – 0.02 | **-0.22 – -0.05** / -0.36 – 0.04 |
| ondansetron | **-0.47 – -0.01** / -1.07 – 0.07 | **-0.46 – -0.06** / -0.93 – 0.09 |
| pantoprazole | **-0.19 – -0.01** / -0.38 – 0.04 | **-0.25 – -0.05** / -0.43 – 0.04 |
| tiotropium bromide | **-0.43 – -0.02** / -0.82 – 0.05 | **-0.44 – -0.07** / -0.81 – 0.08 |
| amoxicillin | - | **-0.37 – -0.03** / -0.75 – 0.1 |
| dexamethasone | - | **-0.3 – 0.00** / -0.63 – 0.14 |
| metoclopramide | - | **-0.31 – -0.01** / -0.63 – 0.12 |
| ramipril | - | **-0.13 – 0.00** / -0.24 – 0.08 |
| rosuvastatin | - | **-0.31 – -0.02** / -0.65 – 0.12 |
| tozinameran | - | **-0.33 – 0.00** / -0.74 – 0.15 |
| venlafaxine | - | **-0.28 – 0.00** / -0.61 – 0.15 |
| **Strong negative predictors** |  |  |
| candesartan | **-0.36 – -0.10 / -0.55 – 0.00** | **-0.38 – -0.16 / -0.54 – -0.04** |

Credibility intervals that do not cover zero are shown in bold text.
