# Supplementary material for: Dealing with adverse drug reactions in the context of polypharmacy using regression models
Source: Sci Rep. 2024 Nov 9;14:27355. doi: 10.1038/s41598-024-78474-4 (PMC11550797; doi:10.1038/s41598-024-78474-4)
Supplement: Supplementary file 4 — Supplementary Material 4 [file 41598_2024_78474_MOESM4_ESM.docx]

**Supplement 4**: Potential negative predictors of regression methods using horseshoe and lasso priors for the outcome ADR “bleeding”

|  | **50%/ 90% credibility intervals** | |
| --- | --- | --- |
|  | **Horseshoe** | **Lasso** |
| **Light negative predictors** |  |  |
| amiodarone | **-0.41 – -0.03** / -0.71 – 0.03 | **-0.40 – -0.08** / -0.65 – 0.07 |
| dexamethasone | **-0.28 – -0.01** / -0.60 – 0.05 | **-0.37 – -0.09** / -0.61 – 0.05 |
| ezetimibe | **-0.23 – -0.01** / -0.47 – 0.04 | **-0.31 – -0.08** / -0.48 – 0.05 |
| furosemide | **-0.13 – 0.00** / -0.28 – 0.05 | **-0.20 – -0.04** / -0.33 – 0.05 |
| insulin human | **-0.16 – 0.00** / -0.35 – 0.05 | **-0.23 – -0.03** / -0.39 – 0.09 |
| insulin glargine | **-0.17 – -0.01** / -0.33 – 0.03 | **-0.24 – -0.06** / -0.37 – 0.04 |
| nitroglycerin | **-0.24 – 0.00** / -0.56 – 0.06 | **-0.35 – -0.07** / -0.57 – 0.05 |
| opipramol | **-0.39 – -0.02** / -0.72 – 0.04 | **-0.44 – -0.12** / -0.70 – 0.03 |
| oxycodone | **-0.24 – -0.01** / -0.48 – 0.05 | **-0.36 – -0.10** / -0.55 – 0.03 |
| ramipril | **-0.09 – 0.00** / -0.17 – 0.03 | **-0.12 – -0.03** / -0.19 – 0.03 |
| xipamide | **-0.26 – -0.03** / -0.45 – 0.03 | **-0.3 – -0.10** / -0.46 – 0.02 |
| zolpidem | **-0.3 – -0.01** / -0.63 – 0.05 | **-0.4 – -0.09** / -0.67 – 0.06 |
| zopiclone | **-0.25 – -0.01** / -0.51 – 0.05 | **-0.34 – -0.09** / -0.55 – 0.04 |
| amoxicillin | **-0.47 – -0.04** / -0.81 – 0.03 | **-** |
| hydrochlorothiazide | **-0.19 – -0.03** / -0.30 – 0.01 | **-** |
| aciclovir | - | **-0.28 – -0.01** / -0.52 – 0.13 |
| atorvastatin | - | **-0.13 – -0.01** / -0.21 – 0.06 |
| carboplatin | - | **-0.24 – 0.00** / -0.46 – 0.14 |
| empagliflozin | - | **-0.26 – -0.02** / -0.48 – 0.12 |
| fluorouracil | - | **-0.30 – -0.04** / -0.55 – 0.10 |
| gemcitabine | - | **-0.36 – -0.05** / -0.64 – 0.08 |
| methotrexate | - | **-0.30 – -0.04** / -0.54 – 0.10 |
| ondansetron | - | **-0.32 – -0.03** / -0.6 – 0.13 |
| risperidone | - | **-0.26 – -0.03** / -0.46 – 0.11 |
| **Strong negative predictors** |  |  |
| bisoprolol | **-0.28 – -0.15 / -0.36 – -0.04** | **-0.28 – -0.16 / -0.36 – -0.07** |
| tozinameran | **-1.62 – -1.00 / -2.17 – -0.57** | **-1.22 – -0.67 / -1.68 – -0.34** |
| amoxicillin | **-** | **-0.51 – -0.19** / **-0.77 – 0.00** |
| hydrochlorothiazide | **-** | **-0.23 – -0.10 / -0.33 – -0.02** |

Credibility intervals that do not cover zero are shown in bold text.
